# Cortisol, DHEA-S, and cortisol/DHEA-S ratio in association with oxidative stress in Korean adults

**DOI:** 10.3389/fendo.2025.1708007

**Published:** 2025-12-15

**Authors:** Sat Byul Park

**Affiliations:** Department of Family Practice and Community Health, Ajou University School of Medicine, Suwon, Republic of Korea

**Keywords:** Cortisol, DHEA-S, cortisol/DHEA-S ratio, oxidative stress, Hemoglobin, GGT

## Abstract

**Background:**

Oxidative stress can result from redox reactions involving hemoglobin (Hb) and nitric oxide (NO), forming toxic peroxynitrite when NO reacts with superoxide during Hb autoxidation. γ-glutamyltranspeptidase (GGT) also contributes to reactive oxygen species generation in the presence of transition metals like iron. The hypothalamic–pituitary–adrenal axis is central to the body’s stress response and immunomodulation. This study aimed to investigate the relationships between cortisol, dehydroepiandrosterone sulphate (DHEA-S), the cortisol/DHEA-S ratio, and oxidative stress markers such as Hb and GGT in a Korean population.

**Methods:**

Data from 1,341 adults collected between January 2018 and March 2023 were analyzed. Sociodemographic and lifestyle data were gathered via questionnaires. Body composition, blood pressure, and metabolic variables, including cortisol and DHEA-S levels, were evaluated.

**Results:**

The mean age of participants was 52.6 ± 11.9 years. Higher cortisol and DHEA-S levels were significantly linked to elevated Hb and GGT levels. Conversely, the cortisol/DHEA-S ratio was negatively correlated with Hb levels. Logistic regression confirmed that higher Hb levels were associated with increased cortisol (P = 0.038) and DHEA-S levels (P < 0.001). The GGT level had no significant association with the cortisol/DHEA-S ratio.

**Conclusions:**

Elevated cortisol and DHEA-S levels are associated with markers of oxidative stress in Korean adults, independent of age and body mass index.

## Introduction

The hypothalamic–pituitary–adrenal (HPA) axis is a critical neuroendocrine system that maintains homeostasis, particularly through its response to stress and its modulation of the immune system (1). Two primary adrenal hormones, cortisol and dehydroepiandrosterone (DHEA), exert opposing effects on immune function; cortisol generally acts as an immunosuppressant, while DHEA and its sulfated form, DHEA-S, often enhance immune function (2). An increased cortisol-to-DHEA ratio has been associated with immunosenescene, age-related decline in immune function (3).

With aging, the HPA axis plays a pivotal role in stress response and immunomodulation (1, 4). The production of sulfated ester (DHEA-S) peaks at around 20 years of age and decreases to only 10–20% of the maximal level by 70 years (5). In contrast, cortisol production remains stable with age (5), resulting in a relative glucocorticoid excess in older adults (6) and a potentially altered stress response.

Stress, defined as perceived threat to homeostasis, involves key components located in the hypothalamus (paraventricular nucleus), anterior pituitary gland, and adrenal glands, collectively forming the HPA axis (7). Studies using gene knockout to modify glucocorticoid receptor expression have demonstrated the receptor’s essential role in survival and various physiological functions, including immunomodulation (8). This is consistent with the fact that glucocorticoids are critical components of the stress response and display anti-inflammatory/immunosuppressive actions (9).

Oxidative stress is associated with redox reactions involving hemoglobin (Hb), nitrite, and nitric oxide (NO), leading to the formation of highly toxic peroxynitrite when NO reacts with superoxide released during Hb autoxidation (10). DHEA-S induces erythropoiesis by acting as a weak androgen, contributing to androgenicity after conversion to testosterone and dihydrotestosterone (11). Testosterone enhances hematopoiesis and participates in several physiological functions, including erythropoiesis (12). The primary function of the respiratory heme protein Hb is to reversibly bind oxygen. It may also be involved in the metabolism and transport of NO. The physiological requirement of these heme proteins to bind oxygen to a redox-active metal can promote oxidative stress reactions. These reactions tend to be uncontrolled and lead to the formation and leakage of reactive oxygen species (ROS), free radicals, and cytotoxic derivatives of Hb. Tissue damage resulting from these “rogue” activities of Hb can lead to a series of pathological complications in certain disease states, or when cell-free Hb is used as blood substitute (13).

The primary role of cellular γ-glutamyltranspeptidase (GGT) is to metabolize extracellular reduced glutathione (GSH), facilitating intracellular GSH synthesis. Recent studies suggest that GGT may also be involved in ROS generation in the presence of iron or other transition metals. Serum GGT is often used as a marker for excessive alcohol consumption or liver diseases, whereas normal-range GGT might serve as an early indicator of oxidative stress (14). Cortisol is released from subcutaneous adipose tissue by 11β-hydroxysteroid dehydrogenase type 1, with increased enzyme expression in obesity likely enhancing local glucocorticoid signaling and contributing to whole-body cortisol regeneration (15). DHEA targets adipocytes, inhibiting preadipocyte proliferation, differentiation, and fat accumulation. Moreover, it stimulates triacylglycerol hydrolysis, enhances glucose uptake, and suppresses cortisol synthesis in adipose tissues. DHEA also alters adipokine expression and secretion in adipocytes (16). This study hypothesized that cortisol and DHEA-S levels and the cortisol/DHEA-S ratio would be positively and/or independently associated with oxidative stress indicators, such as Hb and GGT levels and obesity.

## Materials and methods

### Characteristics of study participants

A total of 1,341 Korean adults aged over 20 years who underwent medical evaluation at Ajou University Hospital between January 2018 and March 2023 were enrolled in this study. Participants taking steroid medication were excluded. The final study population consisted of 1,341 participants (69.4% female). The study protocol was approved by the Institutional Review Board of Ajou University Hospital (AJOUIRB-DB-2024-571), which ensures ethical conduct in studies involving human participants.

### Data collection

Sociodemographic characteristics, smoking status, alcohol consumption, and activity level were assessed using questionnaires. Height and weight were measured using bioelectrical impedance analysis (Inbody 3.0, Biospace, Korea, 2021) after an overnight fast. Body mass index (BMI) was calculated as weight divided by height squared (kg/m^2^) (17). Waist circumference (WC) was measured by a trained nurse at the midpoint between the lower rib and the iliac crest. Blood pressure was measured using a semi-automated blood pressure monitor (TM-2650A; PMS Instruments, Tokyo, Japan) after resting for at least 15 min.

### Blood sample collection

Venous blood samples were collected at 8:00 a.m. after 12-hour overnight fast and 24 h of abstinence from vigorous physical activity. Fasting Hb levels were measured using a DxH 900 analyzer (Beckman Coulter Inc., CA, USA). GGT levels were assessed using an enzymatic colorimetric method with a TBA-200FR analyzer (Toshiba, Tokyo, Japan). Cortisol and DHEA-S levels were assessed using the RIA method (Beckman Coulter Inc. USA). Blood serum was used for the calibration curves and was collected in serum separator clot activator (SST tube). Standard curves were constructed in the correct biological matrix. The limit of detection was 8.60 nM. For repeatability the coefficients of variation were found below or equal to 7.49%. For within-laboratory precision the coefficients of variation were found below or equal to 13.7%. The assay demonstrate to be linear from 12.60 to 2,520 nM. Measurement ranges are 8.60 to approximately 2,000 nM. Standard deviation and coefficient of variation for repeatability were 28.45 nM and 5.38%. Standard deviation and coefficient of variation for within laboratory precision were 24.66 nM and 7.37%.

### Statistics

Student’s t-tests were used to compare the mean values of general characteristics and laboratory results between men and women. Pearson’s correlation analysis was performed to assess the relationship between the serum levels of Hb, GGT, cortisol, DHEA-S, and the cortisol/DHEA-S ratio. Partial correlations were calculated to adjust for age and BMI. Linear and logistic regression analyses were conducted to evaluate the associations between oxidative stress markers and other study parameters. A P-value of less than 0.05 was considered statistically significant. All statistical analyses were formed using SPSS (version 25.0).

## Results

### Study group participant properties

The study included 1,341 individuals: 410 men (30.6%) and 931 women (69.4%). The mean age was 52.6 ± 11.9 years, and the mean BMI was 24.1 ± 4.1 kg/m^2^. The mean cortisol level was 12.7 ± 4.6 μg/dL and mean DHEA-S level was 106.5 ± 81.1 μg/dL. Significant sex differences were observed in several variables, including WC, BMI, and levels of Hb, GGT, cortisol, and DHEA-S (**Table 1**).

**Table 1:** General characteristics of the study participants.

Variable	Male (n=410)	Female (n=931)	P
Age (years)	53.3 ± 12.0	52.3 ± 11.9	0.156
WC (cm)	89.6 ± 10.2	84.4 ± 9.1	<0.001
BMI (kg/m^2^)	25.5 ± 4.2	23.5 ± 3.9	<0.001
SBP (mmHg)	126.2 ± 13.2	120.0 ± 14.0	<0.001
DBP (mmHg)	78.3 ± 9.9	72.7 ± 9.5	<0.001
Hb (g/dL)	15.3 ± 1.28	13.4 ± 1.09	<0.001
GGT (U/L)	42.9 ± 44.9	23.9 ± 28.2	<0.001
FBS (mg/dL)	102.5 ± 22.6	95.2 ± 14.1	<0.001
T-Chol (mg/dL)	195.2 ± 38.9	199.0 ± 37.2	0.265
TG (mg/dL)	155.2 ± 103.3	109.1 ± 56.8	<0.001
HDL-C (mg/dL)	51.5 ± 13.1	63.4 ± 15.1	<0.001
LDL-C (mg/dL)	116.9 ± 36.1	116.3 ± 32.4	0.843
Cortisol (μg/dL)	13.5 ± 4.5	12.3 ± 4.6	<0.001
DHEA-S (μg/dL)	145.8 ± 92.7	89.1 ± 68.7	<0.001
Cortisol/DHEA-S ratio	0.18 ± 0.34	0.28 ± 0.39	<0.001

Values are mean ± SD unless otherwise indicated. BMI, body mass index; SBP, systolic blood pressure; DBP, diastolic blood pressure; Hb, hemoglobin; GGT, γ-glutamyltranspeptidase; FBS, fasting blood sugar; T-Chol, total cholesterol; TG, triglyceride; HDL-C, high density lipoprotein cholesterol; LDL-C, low density lipoprotein cholesterol, DHEA-S; dehydroepiandrosterone-sulfate. Gender difference of continuous variables were compared using *t*-test.

### Correlations of hormones and their ratio with Hb and GGT levels

**Figure 1:**
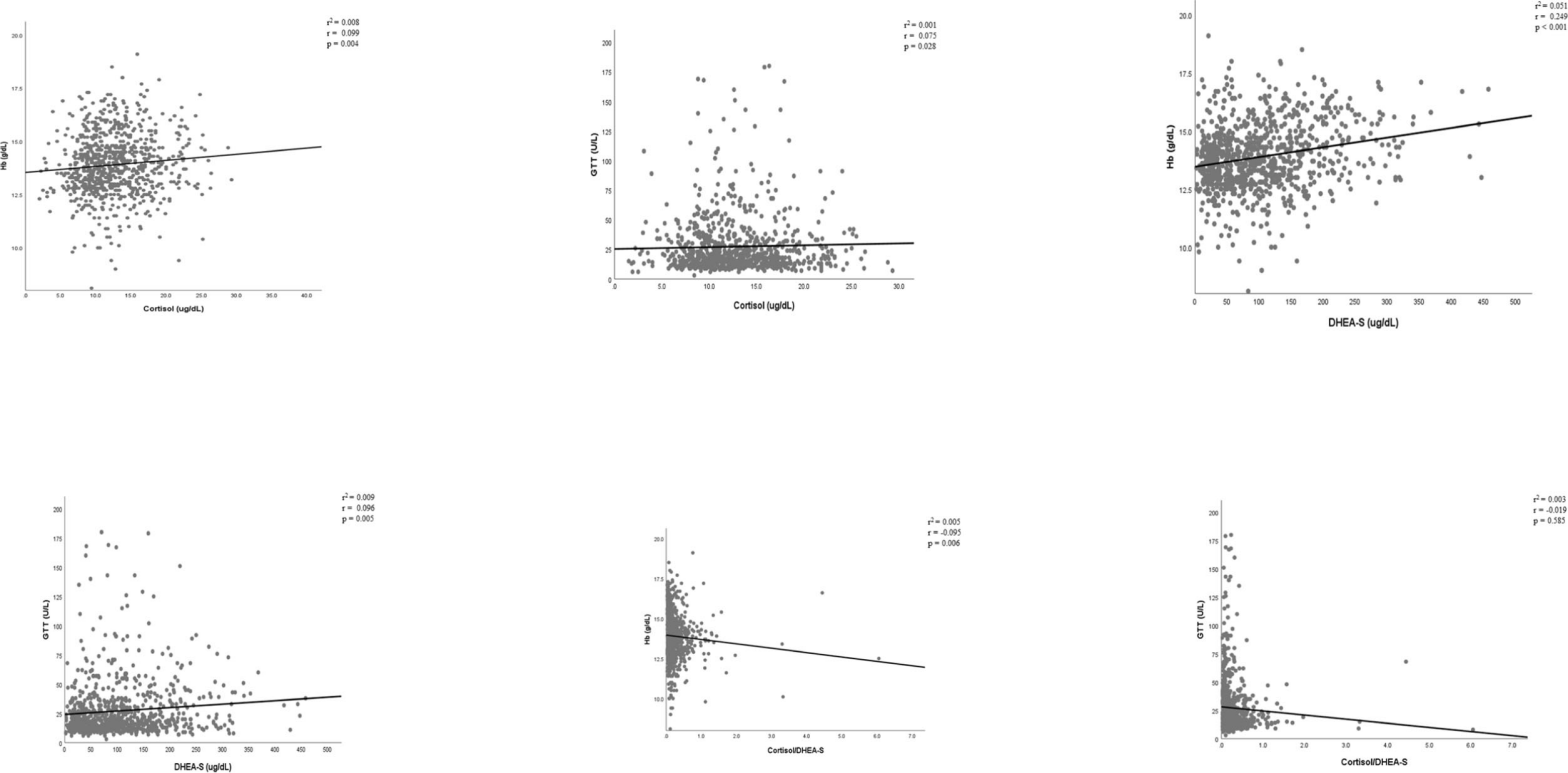
Correlations of hormones and their ratio with Hb and GGT levels.

Linear regression analyses evaluating the association of cortisol level with Hb and GGT levels, adjusting for BMI and age, are shown in **Figure 1**. The results showed significant associations between cortisol, Hb, and GGT levels after adjusting for age and BMI. Elevated cortisol levels were associated with higher Hb (P = 0.004) and GGT levels (P = 0.028).

### Correlations of DHEA-S level with Hb and GGT Levels

The mean DHEA-S level was 145.8 ± 92.7 μg/dL for men and 89.1 ± 68.7 μg/dL for women (**Table 1**). The associations of DHEA-S level with Hb (P<0.001) and GGT levels (P = 0.005) were observed after adjusting for age and BMI.

### Correlations of the cortisol/DHEA-S ratio with Hb and GGT levels

The cortisol/DHEA-S ratio was negatively associated with Hb level (P = 0.006). The association between the cortisol/DHEA-S ratio and GGT level was not significant (P = 0.585). A trend toward an association between the cortisol/DHEA-S ratio and antioxidant effects was observed (P = 0.06).

### Logistic regression for predicting higher Hb levels

Higher cortisol and DHEAS levels were associated with an increased risk of higher Hb levels (P = 0.038 and P<0.001, respectively) after adjusting for age and BMI. However, this effect was not significant for the cortisol/DHEA-S ratio (**Table 2**).

**Table 2:** Logistic regression in the prediction of level of Hb.

Predictor variable	B	SE	P
Cortisol	0.042	0.02	0.038
Age	-0.008	0.008	0.302
BMI	0.012	0.006	0.046
DHEA-S	0.007	0.001	<0.001
Age	0.019	0.009	0.037
BMI	0.01	0.006	0.124
Cortisol/DHEA-S	-0.778	0.458	0.089
Age	-0.002	0.009	0.849
BMI	0.011	0.006	0.063

BMI, body mass index; DHEA-S, dehydroepiandrosterone sulphate.

## Discussion

This study demonstrates a significant association between elevated levels of the HPA axis hormones (cortisol and DHEA-S) and markers of oxidative stress in a large sample of Korean adults, independent of age and BMI. Oxidative stress is defined as an imbalance between free radical damage and antioxidant protection caused by the presence of free radicals or radical-generating agents at concentrations that overwhelm natural radical-blocking or radical-scavenging mechanisms. Oxidative stress induces oxidative damage to biological substances, such as lipids, DNA, and proteins (18). It is linked to various health issues, including inflammation, cellular damage, and aging. Managing stress and maintaining balanced cortisol levels are widely recognized as crucial for overall health.

Both oxidative stress and elevated plasma cortisol levels are recognized risk factors for atherosclerosis and cardiovascular disease (19). The positive association between cortisol and Hb levels aligns with cortisol’s known role in potentially stimulating erythropoietin production during stress, which increases red blood cell production increased red blood cell production. Although elevated cortisol level is not directly associated with an elevated level of GGT, an enzyme involved in glutathione metabolism and amino acid transport, indirect relationships may exist. For example, prolonged stress and chronic cortisol level elevation are often observed in conjunction with lifestyle changes, such as increased alcohol consumption or metabolic syndrome, which are associated with increased GGT levels. Cortisol is understood to indirectly influence liver function through its role in processes such as fat metabolism and glucose production, which may be related to GGT levels (20). This study supports the concept that prolonged activation of the HPA axis, with resulting high cortisol levels, is intertwined with increased oxidative stress.

Similarly, the positive correlation between DHEA-S and both Hb and GGT is a key finding. The relationship with Hb may be explained by the androgenic properties of DHEA-S, which is known to be converted to testosterone and is implicated in stimulating erythropoiesis (11). Research suggests that DHEA and its sulfated form, DHEA-S, may help reduce oxidative stress by enhancing the antioxidant capacity of the body (21). However, the relationship between DHEA-S and oxidative stress is complex and influenced by various factors, including age, health conditions, and overall hormonal balance. In some contexts, altered DHEA-S levels may reflect or contribute to oxidative imbalance. As part of the endocrine system, changes in DHEA-S levels are known to interact with other aspects of metabolism and stress responses, indirectly contributing to oxidative stress dynamics. Also, DHEA-S is understood to modulate the body’s response to stress, indirectly influencing oxidative stress outcomes by interacting with stress hormones and related metabolic changes (22). Studies examining the relationship between sex hormone concentrations and inflammatory markers have shown inconsistent results, often due to small sample sizes and cross-sectional designs. Although in some studies, different sex hormones were not correlated with inflammatory biomarkers (23–25), positive correlations were observed between DHEA-S level and high-sensitivity C-reactive protein (hs-CRP) level (26). A Turkish study on patients with obesity reported a positive correlation between DHEA-S levels and BMI in both sexes (27).

A significant positive association between DHEA-S, Hb, and hematocrit levels has been observed in men (28). Although the mechanism remains unclear, DHEA-S may induce erythropoiesis by acting as a weak androgen. DHEA-S is a weak androgen that contributes to androgenicity, mainly after peripheral conversion to the more potent androgens testosterone and dihydrotestosterone (10). Hb in red blood cells continuously undergoes redox reactions with oxygen, producing superoxide and hydrogen peroxide (29).

Elevated serum level of GGT, a marker of liver dysfunction, has also been suggested as an early marker of oxidative stress and inflammation (14). GGT level correlates positively with hs-CRP and fibrinogen levels (30, 31) and inversely with antioxidant levels (32). Haring et al. (33) suggested divergent mechanisms by which sex hormone levels are involved in the atherosclerotic process, showing consistent associations with the prothrombotic marker, fibrinogen, and the oxidative stress marker, GGT. Elevated GGT levels have been associated with changes in hormonal profiles, including that of DHEA-S. For instance, liver dysfunction is known to affect steroid hormone metabolism, potentially leading to altered DHEA-S levels. Conversely, high DHEA-S levels may influence liver function and GGT levels due to the effects of hormones on metabolism (33). These findings challenge the uniformly protective role of DHEA-s and suggest its effect on oxidative balance may be context-dependent. Further studies are needed to clarify the association between sex hormones and GGT.

Interestingly, the cortisol/DHEA-S ratio was not significantly associated with GGT levels. This non-significant result is also informative. It may suggest that the individual effects of cortisol and DHEA-S on liver enzyme activity (reflected by GGT) are more direct or potent than their combined ratio. The balance between these two hormones might be more critical for systemic processes like immune function, as suggested by immunosenescence research (34), rather than for specific enzymatic markers of oxidative stress like GGT. This highlights the complexity of these hormonal interactions and indicates that different biomarkers of oxidative stress may be influenced by different aspects of HPA axis function. Therefore, the actions of the two hormones in the immune system are interdependent (35).

This study has some limitations. First, the sample comprised patients who visited a University Hospital, which may restrict the generalizability of our findings. Second, the cross-sectional design precludes causal conclusions regarding the relationship between cortisol and DHEA-S levels with Hb and GGT levels. Longitudinal studies are needed to determine whether circulating cortisol and DHEAS levels are causally linked to an increased risk of oxidative stress. Further research into the underlying mechanisms could lead to new therapeutic approaches for reducing oxidative stress.

## Conclusion

This study provides strong evidence that elevated plasma cortisol and DHEA-S levels are independently associated with increased markers of oxidative stress (Hb and GGT) in Korean adults. These relationships persist after controlling for age and BMI, thereby enhancing understanding of the important link between HPA axis activity and oxidative balance. The findings contribute to a deeper understanding of the physiological impact of stress-related hormones and highlight the need for further research into the mechanisms connecting the endocrine system to oxidative stress pathways.

## Data Availability

The original contributions presented in the study are included in the article/supplementary material. Further inquiries can be directed to the corresponding author.
